# Sensor-Driven Localization of Airborne Contaminant Sources via the Sandpile–Advection Model and (1 + 1)-Evolution Strategy

**DOI:** 10.3390/s25196215

**Published:** 2025-10-07

**Authors:** Miroslaw Szaban, Anna Wawrzynczak

**Affiliations:** 1 Institute of Computer Science, University of Siedlce, 08-110 Siedlce, Poland; anna.wawrzynczak-szaban@uws.edu.pl; 2 National Centre for Nuclear Research, 05-400 Otwock, Poland

**Keywords:** Sandpile model, evolution strategy (1 + 1), optimization process, airborne contaminant source, inverse methods

## Abstract

The primary aim of this study is to develop an effective decision-support system for managing crises related to the release of hazardous airborne substances. Such incidents, which can arise from industrial accidents or intentional releases, necessitate the rapid identification of contaminant sources to enable timely response measures. This work focuses on a novel approach that integrates a modified Sandpile model with advection and employs the (1 + 1)-Evolution Strategy to solve the inverse problem of source localization. The initial section of this paper reviews existing methods for simulating atmospheric dispersion and reconstructing source locations. In the following sections, we describe the architecture of the proposed system, the modeling assumptions, and the experimental framework. A key feature of the method presented here is its reliance solely on concentration measurements obtained from a distributed network of sensors, eliminating the need for prior knowledge of the source location, release time, or emission strength. The system was validated through a two-stage process using synthetic data generated by a Gaussian dispersion model. Preliminary experiments were conducted to support model calibration and refinement, followed by formal tests to evaluate localization accuracy and robustness. Each test case was completed in under 20 min on a standard laptop, demonstrating the algorithm’s high computational efficiency. The results confirm that the proposed (1 + 1)-ES Sandpile model can effectively reconstruct source parameters, staying within the resolution limits of the sensor grid. The system’s speed, simplicity, and reliance exclusively on sensor data make it a promising solution for real-time environmental monitoring and emergency response applications.

## 1. Introduction

The emission and storage of toxic substances pose an ongoing risk of accidental or intentional release into the atmosphere, which can have severe consequences for human health and the environment. Airborne pollution remains a critical area of research due to its potential to affect large geographic areas and densely populated urban centers, particularly when released materials are hazardous or difficult to detect. Such releases often occur due to uncontrolled leaks in industrial storage or transport systems. In other cases, they may be intentional, aimed at causing harm or public disruption. Regardless of the cause, early detection and characterization of these events are essential for a timely and effective response. Sensor systems are crucial for detecting airborne contaminants. Modern sensor networks that can measure chemical concentrations in real time are vital for identifying the presence of toxic substances in the atmosphere. When combined with atmospheric dispersion models, sensor data facilitate accurate tracking of contamination plumes and assist in estimating the source term—specifically, identifying the location, timing, and intensity of the release. The most challenging and dangerous situations arise when elevated concentrations of an unknown contaminant are detected without prior information about its source. In these cases, rapid analysis of sensor data, paired with model-based inference, can help estimate the release coordinates and emission rate. This capability is essential for initiating containment measures, issuing public safety alerts, and preventing the spread of hazardous materials.

The literature includes numerous studies focused on locating the sources of atmospheric contamination using data collected from distributed sensors. Existing algorithms for this task can be divided into two main categories: The first category employs a backward approach, designed for open areas or problems on a continental scale. The second category uses a forward approach, where various parameters of a dispersion model, including the source location, are sampled to identify the one that minimizes the distance between the model outputs and the measurements from the sensors within the specified spatial domain. In our approach, the redistribution mechanism follows a forward and stepwise propagation. This inverse problem does not have a unique analytical solution, but it can be analyzed within probabilistic frameworks such as the Bayesian approach, which treats all variables of interest as random variables (e.g., [1,2]). A comprehensive literature review of previous works addressing the inverse problem of atmospheric contaminant releases can be found in sources like [3].

Identifying the source of an atmospheric contaminant release is a computationally intensive task. Traditional methods often require multiple simulations using complex dispersion models, with each run potentially taking from minutes to days. This significant computational demand poses a challenge in emergency situations that necessitate quick source localization based on data from sensor networks. To address prolonged computation times, artificial neural networks have been proposed as an alternative, especially in urban environments (e.g., [4]). However, the effectiveness of source reconstruction relies not only on the dispersion model used but also on the efficiency of the algorithm employed to search the parameter space to align model outputs with sensor measurements.

Various optimization algorithms, including nature-inspired techniques, have been applied to the problem of source reconstruction. A notable recent development is the Layered Algorithm, which utilizes a two-dimensional, three-state cellular automaton (CA) for classification [5]. This method organizes sensor measurements by magnitude into distinct data layers, allowing the CA to effectively identify the probable source location.

In [6], the author provides a thorough review of significant advancements in unmanned ground-based mobile sensing network configurations and autonomous data acquisition strategies utilized for localizing gaseous plume sources.

The issue of identifying contaminant sources has been addressed in previous studies [7,8,9]. These studies focused on contamination in water distribution networks and employed a methodology that combines a machine learning algorithm with an optimization algorithm. Additionally, the problem of leakages from sewage networks, which can lead to the contamination of groundwater reservoirs, was examined in [10].

This paper centers on the Sandpile algorithm, which has been explored for identifying the source of an atmospheric contaminant release. A preliminary study introduced a simplistic model paired with the Generalized Extremal Optimization algorithm; however, it notably omitted the advection mechanism, limiting its physical realism [11].

In this paper, we build upon that research by significantly expanding it and, most importantly, by incorporating the advection mechanism. This enhancement enables our proposed model to more accurately reflect real-world data. We present a novel tool for identifying the sources of airborne contamination, utilizing data gathered from a distributed network of sensors. Our approach adapts the Sandpile model, traditionally used to simulate self-organized criticality, to represent the transport and dispersion of airborne pollutants within a specified spatial domain. The primary aim is to assess the feasibility of applying the Sandpile model as a simplified yet effective method for simulating the movement of contaminants in the atmosphere.

The structure of this paper is as follows: Section 2 introduces the fundamentals of the Sandpile model and explains its relevance in modeling atmospheric pollution. Section 3 discusses how cellular automata are employed to implement the simulation framework. Section 4 describes the (1 + 1)-Evolution Strategy (ES) algorithm, which is used to optimize the source prediction process. Section 5 briefly explores the concept of advection as an important transport mechanism. Section 6 provides an overview of the Gaussian dispersion model, which generates synthetic reference data for validation purposes. Section 7 presents the simulation results and evaluates the performance of the proposed method. Finally, Section 8 concludes the paper and suggests directions for future research.

## 2. Characteristics of the Sandpile Model

The Sandpile model is a well-established deterministic framework that helps in studying self-organizing criticality. This model was proposed by Bak, Tang, and Wiesenfeld in 1987 (see [12,13,14]). In this framework, the steady state (or critical state) eventually collapses at a certain point in time. The simplest version of the Sandpile model starts with a single column configuration. At each step, if a column has at least two more grains than its right-hand neighbor, it transfers one grain to that neighbor. It has been proven (in [15,16]) that this model converges to a single configuration where the evolutionary rule can no longer be applied to any column. This is known as the fixed point. All possible arrangements that arise from the initial column configuration through the application of the evolution rule can be characterized within a two-dimensional grid [17].

To illustrate the Sandpile model, we can use a simple mental image involving grains of sand [18]. Imagine a pile of sand on a small table. Dropping an additional grain onto the pile can trigger avalanches, causing grains to slide down the slopes. The dynamics of these avalanches depends on the steepness of the slope. As the avalanche occurs, the sand settles somewhere on the table. If the avalanche continues, some grains may fall off the edge of the table. On average, adding one grain to the pile will increase the slope. Over time, as the grains spread, the slope evolves into a critical state. At this point, dropping a single grain onto the pile can result in a large avalanche. This thought experiment illustrates that the critical condition is highly sensitive to stimuli; even a small change—whether internal or external—can lead to significant effects [19].

In the initial stage, we define a class of graphs G=(V∪{s},E) on which the model is based. The graph *G* must be finite, undirected, connected, and loopless. It may contain multiple edges and include a distinctive terminal vertex. The set of graphs is denoted as *G*. The notation u∼v indicates adjacency in *G*, meaning {u,v}∈E.

**Figure 1:**
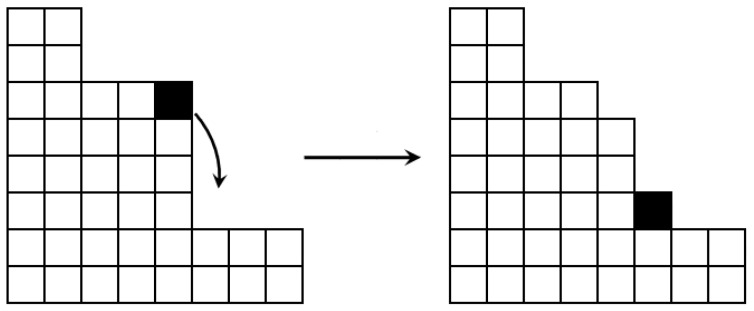
The avalancheoperation of the Sandpile model. Dropping grains creates a slope; after crossing the threshold equal to 3, the slope falls (avalanche effect) [17].

The configuration of the Sandpile *G* model is represented by the vector $\eta =\left(\eta_{\nu },\nu \in V\right)\in Z_{+}^{\left|V\right|}$. The number $\eta_{\nu }$ indicates the number of sand grains at the vertex ν in the configuration η. When this number exceeds a certain threshold, the vertex is considered to be unstable and will distribute one grain of sand to each of its neighbors (see Figure 1). The probability of a neighboring vertex receiving a grain from an unstable vertex is drawn from the interval p∈(0,1). The terminal vertex plays a special role, as it can accept an infinite number of grains and will never become unstable [13]. When the local slope of the stack—resulting from the height difference between the top and the adjacent vertex—exceeds a certain threshold, the grains will redistribute. This process allows one grain to fall onto another pile. It occurs by reducing one pile’s height while increasing adjacent piles’ height. In the subsequent iteration of pouring sand, the local threshold is recalibrated. This procedure continues until all vertices reach a stable configuration. At this point, a new grain is introduced from the left side. The configuration $\eta =(\eta_{\nu },\nu \in V)$ is considered to be stable when $\eta_{\nu }\leq d^{G}\left(\nu \right)$, where $d^{G}\left(\nu \right)$ represents the degree of vertex ν in graph *G* [13,20].

In our study, we employed an asynchronous sequential approach, as the dropping of grains at a single point can trigger an avalanche. This avalanche then induces changes at multiple points, ultimately leading to a stable state in the Sandpile model used in cellular automata (CA). During the avalanche, the sand grains move downward toward the base of the pile. However, if the grains can fall in multiple directions, the direction chosen is based on a probability proportional to the height of the slope in that direction.

The Sandpile model is highly versatile and can be easily adapted to various research problems. It can be implemented in one or two dimensions, with open, closed, or infinite boundaries. This model is widely used in fields such as physics, economics, mathematics, and theoretical computer science [21,22,23,24,25]. Its applications range from cellular automata and information systems to earthquake calculations [26], studies of river sediments [27], the spread of forest fires [28,29,30,31], investigations of the Earth’s magnetosphere [32], studies of precipitation distribution [20], diffusion problems (such as those involving lattice gas and lattice Boltzmann models) [33], social sciences [34], neuroscience [35], and the study of consciousness [36], as well as theoretical mathematical investigations on the application of renormalization group methods [37,38].

## 3. Two-Dimensional CA Approach for Localization Model

CAs and their potential for efficiently performing complex computations were described by S. Wolfram in [39]. This paper focuses on two-dimensional CAs. A cellular automaton is represented as a rectangular grid of X×Y cells, where each cell can assume one of *k* possible states. After establishing the initial states of all cells (that is, the initial configuration of the cellular automaton), each cell updates its state according to a transition function TF, which is based on the states of neighboring cells.

This study utilizes a finite cellular automaton with stable boundary conditions. State transitions in the cellular automaton occur asynchronously. The transition function discussed in this paper is derived from the Sandpile model, which is applied to the cellular automaton grid. In this context, the cells of the cellular automaton simulate the evolution of sand grains in the Sandpile model.

It is assumed that the contaminant distribution spans the region [0,10,000]×[0,10,000] meters. Consequently, the data space must be mapped from [0,10,000]×[0,10,000] onto the grid of X×Y cells. For simplicity, this paper assumes a square grid with X=100 (see also [11]).

## 4. Localization Model Based on the (1 + 1)-ES-Sandpile Model

Evolution strategies (ESs) are one of the four primary categories of evolutionary algorithms. A key characteristic of ESs is their unique method for adapting the mutation step size. If new solutions do not improve upon previous ones, the algorithm dynamically adjusts the mutation range. Another notable feature of ESs is that a well-adapted individual can proceed to the next generation without any modifications.

Evolution strategies can be classified into several types (see [40,41,42,43,44]). One example is the (1 + 1)-ES, which generates a single offspring from one parent and selects the better-adapted individual to move on to the next generation, as illustrated in Algorithm 1. Over time, new variants of evolution strategies have developed, such as the (μ,λ) and (μ+λ) strategies. These types are primarily utilized for problems where the encoding can be easily represented as a vector of floating-point numbers.

Algorithm 1 illustrates the procedure for the (1 + 1)-Evolution Strategy. In this context, σ represents the mutation range parameter, and N(0, 1) denotes the normal distribution. Additionally, Φ(σ) is the self-adaptive mutation range algorithm presented in Algorithm 2.

Algorithm 1 outlines the operation of the (1 + 1)-ES process. This is an iterative algorithm that involves a series of actions repeated in each iteration *t*, as detailed in lines 4–15 of Algorithm 1. The results obtained from these actions lead to cascading conditional statements, which trigger the execution of specific paths within the algorithm (as seen in lines 7–13 of Algorithm 1). The algorithm operates in the following manner:

The first step (line 2) is to generate a random solution $X^{t}$ of the algorithm, which must satisfy the formal assumptions. In all types of evolution strategies, individuals are represented by a pair of vectors: *X* and σ. The solution vector $X=(X_{1},\;X_{2},\;\ldots ,\;X_{n})$ represents an individual within an *n*-dimensional solution space. The mutation step-size vector $\sigma =(\sigma_{1},\;\sigma_{2},\;\ldots ,\;\sigma_{n})$ contains values where $\sigma_{j}$ is used to mutate the gene $X_{j}$, with *j* ranging from 1 to *n*. The mutation process affects both vectors. First, the step-size vector σ is mutated using the parameters $c_{d}$ and $c_{i}$ (as detailed in Algorithm 2). After this adjustment, the solution vector *X* is mutated using the newly modified step size σ (as outlined in Algorithm 1). The next step (line 3) is to evaluate this solution, $F(X^{t})$, according to Equation (1). Subsequently, the solution $X^{t}$ is analyzed and modified in the following iterations of the algorithm (lines 4–15). A mutation operation occurs (line 5), creating a new solution $Y^{t}$. This new solution is produced by modifying the solution $X^{t}$ by adding the product of the mutation range parameter (σ) and a value drawn from a normal distribution N(0,1). In the next step, the newly created solution $Y^{t}$ is evaluated (line 6). If solution $Y^{t}$ is evaluated higher, we have achieved a better result (marked as success, line 7). In this case, solution $Y^{t}$ is formally implemented as $X^{t}=Y^{t}$ (line 8) and advances to the next iteration. Otherwise (if we do not observe success), solution $X^{t}$ remains for the next iteration (line 11) as the best solution found so far. The algorithm terminates when all predefined iterations have been completed (line 4). The final result is the solution $X^{t}$, evaluated as $F(X^{t})$.

In Algorithm 1, we observe the self-adaptation of the mutation range parameter on lines 9 and 12, which is detailed in Algorithm 2.
**Algorithm 1** Evolutionary strategy (1 + 1)1:t=02:create $X^{t}$3:evaluate $X^{t}$→ calculate $F(X^{t})$4:**while** termination condition NOT TRUE (t=Max) **do**5:    $Y^{t}=X^{t}+\sigma *N\left(0,\;1\right)$ (mutation of $X^{t}$)6:    evaluate $Y^{t}$→ calculate $F(Y^{t})$7:    **if** $F(Y^{t})$ is better than $F(X^{t})$ (success) **then**8:        $\{X^{t}=Y^{t}$9:        σ=Φ(σ)}10:    **else**11:        $\{X^{t}=X^{t}$12:        σ=Φ(σ)}13:    **end if**14:    t=t+115:**end while**16:return $X^{t}$ (effect of the algorithm work)

**Algorithm 2** Self-adaptive mutation range algorithm for evolutionary strategy (1 + 1).
1:calculate $\phi \left(t\right)=\frac{\mathrm{number}\;\mathrm{of}\;\mathrm{successes}\;\mathrm{in}\;\mathrm{iteration}\;t}{t}$2:**if** $\phi \left(t\right)<\frac{1}{5}$ **then**3:    $\left\{\Phi \left(\sigma \right)=c_{d}*\sigma \right\}$4:
**end if**
5:**if** $\phi \left(t\right)=\frac{1}{5}$ **then**6:    {Φ(σ)=σ}7:
**end if**
8:**if** $\phi \left(t\right)>\frac{1}{5}$ **then**9:    $\left\{\Phi \left(\sigma \right)=c_{i}*\sigma \right\}$10:
**end if**



The function ϕ(t) adjusts the mutation range parameter, sigma, based on the number of successes in subsequent iterations (see Algorithm 2, line 1). This allows for an automatic and flexible selection of the search space range, depending on whether the algorithm discovers new, better solutions or becomes stuck in a local extremum. The current sigma value is modified by multiplying it by the constants $c_{d}$ and $c_{i}$ (lines 3 and 9), depending on whether specific conditions (lines 2 and 8) are met.

The suggested constants $c_{d}=0.82$ and $c_{i}=\frac{1}{c_{d}}=1.22$ are based on recommendations by Rechenberg and Schwefel. The $\frac{1}{5}$ success rule, represented as ϕ(t), and its corresponding value were initially proposed by Rechenberg following his studies on multidimensional functions. The scaling factors used to adjust the mutation step size—specifically for increasing ($c_{i}$) or decreasing ($c_{d}$) the step—were derived experimentally and are standard recommendations by Schwefel (see [45]).

In the (1 + 1)-Evolution Strategy algorithm, the fitness function is computed as the sum of the relative differences between the results of the Sandpile model and the contaminant concentrations at sensor locations. The difference at each point is calculated using the following formula (based on [11,46]):

(1) $$F\left(C_{j}^{M},\;C_{j}^{E}\right)=\sum_{j=1}^{N}\frac{\log\left(C_{j}^{M}\right)-\log\left(C_{j}^{E}\right)}{\log(C_{j}^{M})},$$

where $C_{j}^{M}$ is the concentration value at the *j*-th sensor location, $C_{j}^{E}$ is the concentration value estimated by the Sandpile model at the *j*-th sensor location, and *N* is the total number of sensors.

There is a significant disparity in the output scales: the Gaussian model produces low sensor concentrations (values much lower than 1), while the Sandpile model results in accumulated grain counts in the order of $10^{3}$. Applying a logarithm effectively normalizes these values to a comparable order of magnitude. Furthermore, our analysis assumes that the data has been pre-processed; we did not consider scenarios involving noisy or incomplete data, as we presumed that the data had already been corrected and denoised.

If the output of the Sandpile model is equal to 0, it is treated as 1 $\times \;10^{-200}$ to facilitate logarithm calculations. The final evaluation function is the sum of the differences from all of the considered sensor positions. This evaluation function tends toward a minimum; thus, a smaller value of the obtained difference indicates a better match between the Sandpile model and the target concentrations at the sensors.

The (1 + 1)-Evolution Strategy is quite sensitive to local minima, prompting the search for more effective solutions. This search has led to the development of various strategies, including (1+λ), (μ+λ), and (μ,λ).

## 5. Advection in the Sandpile Model

In the basic Sandpile model, a point is selected for the addition of successive grains of sand. When a grain falls from a certain height, its trajectory is a straight line from the release point to the top of the grain column located directly beneath it.

In the enhanced version proposed in this paper, wind advection plays a significant role in influencing the trajectory of falling grains. The landing position of each grain is determined by mathematical equations that describe the dynamics of particle movement in the air. In this context, the speed and direction of the wind are crucial, highlighting their essential role in shaping the grain’s descent and final location.

**Figure 2:**
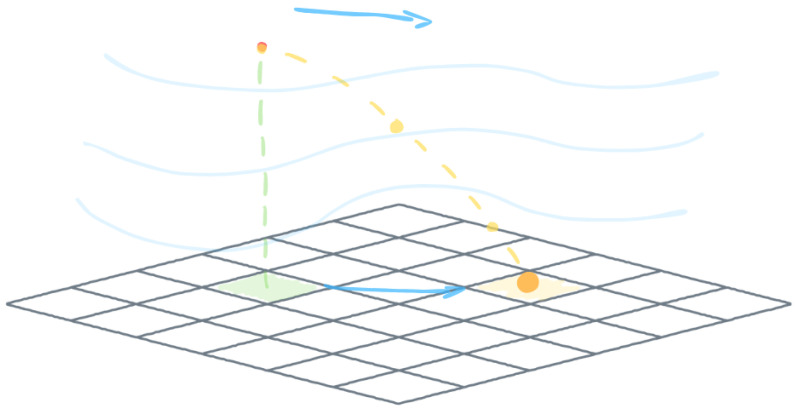
A schematic diagram illustrating the falling trajectory of a sand grain influenced by wind. Source: [47].

Figure 2 provides a conceptual diagram of a sand grain’s trajectory as it falls under the influence of wind. The red dot indicates the release point, while the green path and cell show where the grain would land in the absence of wind. The blue line represents the wind flow, and the yellow path illustrates the grain’s trajectory as influenced by the wind, along with its final landing cell.

Our model, developed within our system, utilizes a cellular automaton approach. Each cell in the grid represents the height of a grain pile, which is determined by the number and size of the grains it contains. When a grain falls onto the peak of a column at coordinates (X, Y), we check its four neighboring cells: (X + 1 , Y), (X − 1 , Y), (X , Y + 1), and (X , Y− 1).

If the height difference between the selected peak and any of its neighbors is no more than three grains, the grain remains at the current peak, and its height increases by the size of the new grain (see [17]). However, if the height difference between the selected peak and a neighboring cell equals the height of four grains, a neighboring peak is randomly chosen for the grain to slide onto. During this process, neighboring peaks that are the same height or higher are excluded from selection. The probability of the grain sliding onto a chosen neighbor increases with the height difference. This cascading movement continues recursively until all grain columns reach a stable state.

The model’s parameters include the coordinates of the grid’s starting point and height, as well as the direction and speed of the wind, which influence the movement of the falling grains. The formula used to calculate the height of a grain at given coordinates, taking into account the wind direction and speed, is as follows:

(2) $$Z=Z_{0}-\frac{g\cdot \sqrt{\frac{X^{2}s_{x}^{2}}{{\left({\mathrm{grad}}_{x}\right)}^{4}}+\frac{Y^{2}s_{y}^{2}}{{\left({\mathrm{grad}}_{y}\right)}^{4}}}}{2v_{0}^{2}},$$

where

- *Z*—The height coordinate of a falling grain at a current planar (X,Y) position in the CA grid;
- $Z_{0}$—The height coordinate of the grain release point $\left(X_{0},Y_{0},Z_{0}\right)$ in the CA grid;
- *g*—Acceleration due to gravity ($9.81\frac{m}{s^{2}}$);
- X,Y—Cell coordinates in the CA grid (current planar position of a falling grain);
- $s_{x},s_{y}$—Scaling factors transforming the planar position (X,Y) in the CA grid to real-world coordinates;
- ${\mathrm{grad}}_{x},\;{\mathrm{grad}}_{y}$—Wind gradient in the CA grid as a vector [▵X,▵Y], where ▵X,▵Y∈[−1;1], which determines the direction and speed of the wind in the CA grid;
- $v_{0}$—Wind speed ($\frac{m}{s}$).

During the simulation, the altitude of a grain is determined by the wind direction gradient for cells within its trajectory. If the grain’s altitude in the next cell is lower than or equal to the altitude of existing grain vertices in that cell— or if it falls below the grid (i.e., z<0)— the grain is deposited onto the vertex in the current cell. For example, if a grain is over the cell (X, Y) and the wind gradient [1, 1] indicates that it will move to cell (X+1, Y+1), we calculate the grain’s altitude at this new location. If this altitude is less than or equal to the vertex height at (X+1, Y+1), or if it is below zero, the grain will be deposited at the vertex of the current cell (X,Y).

**Figure 4:**
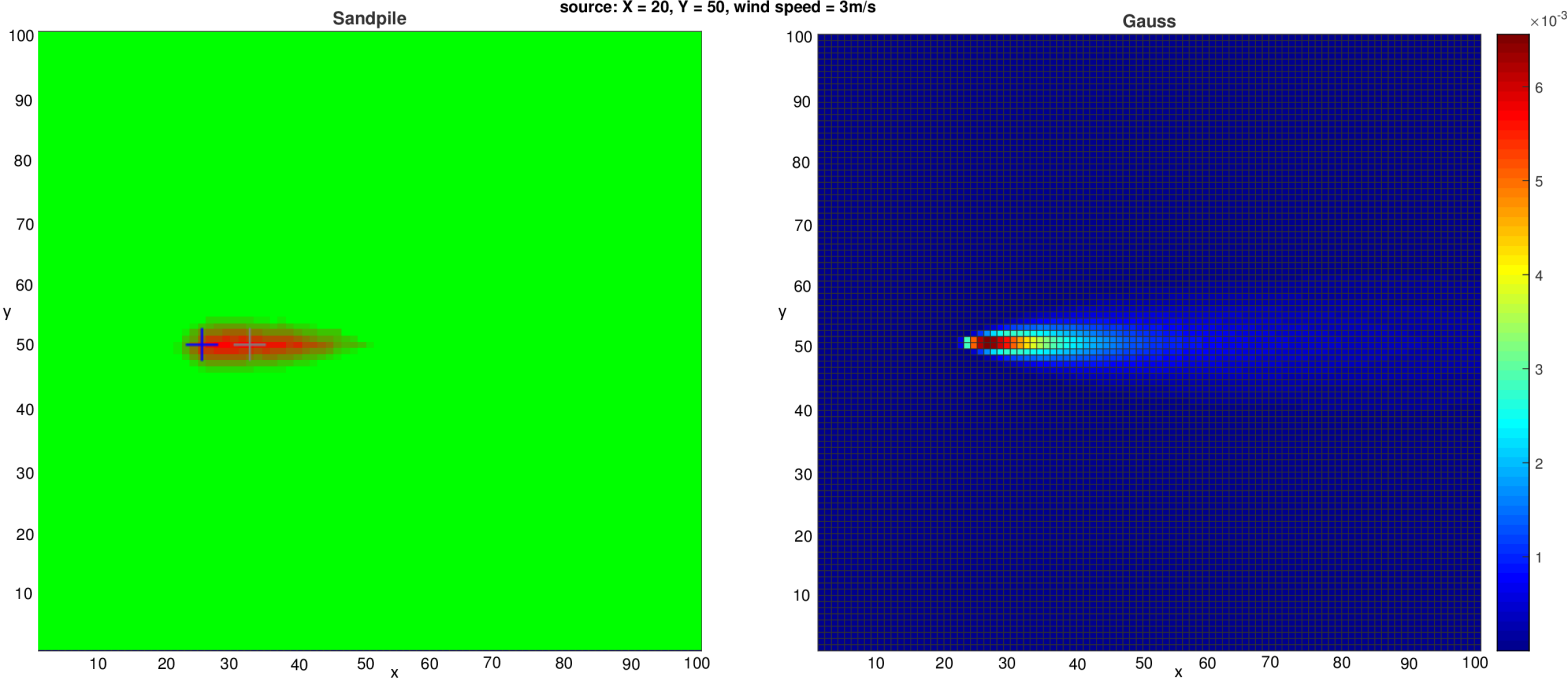
Heat maps illustrating (**left**) the typical distribution of 1000 sand grains in the Sandpile model with advection and (**right**) the contaminant concentration field obtained from the Gaussian dispersion model with an emission rate of $5000\frac{g}{s}$. In both cases, the setup assumes a wind speed of $3\frac{m}{s}$ directed along the *x*-axis and a source located at (20,50).

An example outcome from our model (which includes advection) is shown in the left panel of Figure 4.

## 6. Assessing the Suitability of the (1 + 1)-ES Sandpile Model for Airborne Contaminant Source Localization

### 6.1. Generation of Synthetic Data for Model Evaluation

This chapter examines the applicability of the (1 + 1)-ES-Sandpile model for localizing airborne contaminant sources. To evaluate the effectiveness of the proposed method, synthetic concentration data were generated across the simulation domain using the well-established Gaussian dispersion model (e.g., [48]). This controlled setup allows for a systematic assessment of the algorithm’s ability to accurately identify source locations under simplified yet representative atmospheric conditions.

The Gaussian plume model is one of the most widely utilized methods in air pollution research. It provides a simplified analytical representation of the three-dimensional concentration field produced by a continuous point source under stationary meteorological and emission conditions. Although it is based on several simplifying assumptions, the model remains popular due to its robustness and ease of use. Under uniform and steady wind conditions, the concentration $C(\tilde{x},\tilde{y},z)$ of a contaminant (measured in $\frac{\mu g}{m^{3}}$) at a specific location defined by $\tilde{x}$ meters downwind from the source, $\tilde{y}$ meters laterally from the plume centerline, and *z* meters above ground level can be expressed as follows:

(3) $$C\left(\tilde{x},\tilde{y},z\right)=\frac{Q}{2\pi \sigma_{y}\sigma_{z}U}\exp\left[-\frac{1}{2}{\left(\frac{\tilde{y}}{\sigma_{y}}\right)}^{2}\right]\times \left\{\exp\left[-\frac{1}{2}{\left(\frac{z-H}{\sigma_{z}}\right)}^{2}\right]+\exp\left[-\frac{1}{2}{\left(\frac{z+H}{\sigma_{z}}\right)}^{2}\right]\right\},$$

where *U* denotes the wind speed along the *x*-axis, *Q* is the source strength or emission rate, and *H* is the effective release height, defined as the sum of the physical release height $\tilde{H}$ and the plume rise *h* ($H=\tilde{H}+h$). The dispersion parameters $\sigma_{y}$ and $\sigma_{z}$, representing the standard deviations of the concentration distribution in the crosswind and vertical directions, respectively, are empirical functions of $\tilde{x}$. These parameters depend on the atmospheric stability conditions as established by Pasquill and Gifford (e.g., [48]).

A Gaussian dispersion plume model was used to generate a map of the spread of the contaminant in a specific area. We limited the diffusion to stability class C for the urban environment (using Pasquill-type stability for rural areas; see the classification below and Table 1). The release rate was assumed to vary over time within the range $Q\in \langle 1000\frac{g}{s},10,000\frac{g}{s}\rangle$, which caused changes in the concentrations recorded by the sensors in subsequent time intervals. The wind was directed along the x-axis, with an average speed of $\left(5\;\frac{m}{s}\right)$.

The Pasquill–Gifford stability classes are labeled from A to G, where

A Extremely Unstable—Highly turbulent conditions;
B Moderately Unstable—Very turbulent;
C Slightly Unstable—Less turbulent than B;
D Neutral—Very little or no turbulence;
E Slightly Stable—Stable conditions with some turbulence;
F Moderately Stable—Stable conditions with low turbulence;
G Extremely Stable—The most stable conditions, with very little turbulence.

Table 1 displays the distribution of Pasquill–Gifford stability classes. These classes are determined by factors such as surface wind speed, daytime incoming solar radiation, and nighttime cloud cover. The classification scheme was originally developed by Pasquill [49] and was later modified by Turner [50] and others [51,52].

**Figure 3:**
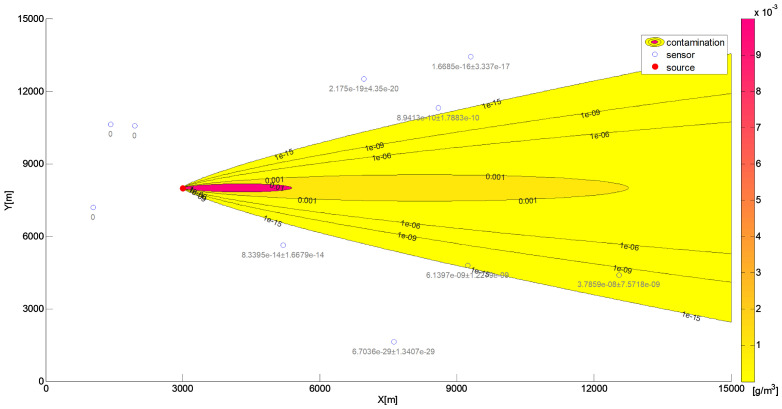
The source location of the sample release and the spatial distribution of concentrations at ten sensor positions within the sample domain for the Gaussian model.

The spatial distribution of the contaminant within the simulation domain is shown in Figure 3. A corresponding sample of the contaminant field, mapped onto the computational grid, is presented in the right panel of Figure 4. For this analysis, the physical domain [0,10,000]×[0,10,000] m was discretized into a 100×100 grid, which served as the input representation for the (1 + 1)-ES-Sandpile model.

**Figure 5:**
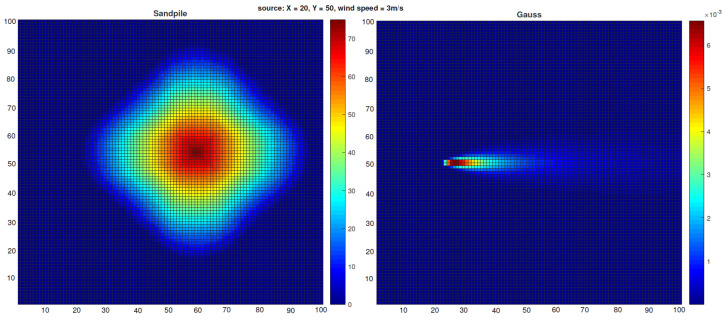
Heat maps illustrating (**left**) the typical distribution of $10^{5}$ sand grains in the simple Sandpile model without advection and (**right**) the contaminant concentration field obtained from the Gaussian dispersion model with an emission rate of $5000\frac{g}{s}$. In both cases, the setup assumes a wind speed of $3\frac{m}{s}$ directed along the *x*-axis and a source located at (20,50). Source: [11].

Figure 4 shows that the contaminant distributions generated by both the Gaussian plume model and the Sandpile model with the proper parameter configuration are nearly identical. This similarity is consistent with the expected behavior of the Sandpile model when advection properties are applied with the same parameters. The results suggest that the Sandpile model is a promising tool for estimating Gaussian distributions and, by extension, for modeling airborne contaminant dispersion. Furthermore, subsequent experiments demonstrate the effectiveness of our advection-enhanced Sandpile model in reconstructing the source location of the released substance. In the case of the Sandpile model without advection (see Figure 5), we can observe the typical symmetric shape of the pile presented in [11]. This shape remains largely unchanged even with varying wind speeds or directions in the Gaussian plume model.

The opposite approach was demonstrated in [53], where the Gaussian plume model was used as a tool to find the location of the gas source in the experimental environment of the authors.

### 6.2. Testing Framework and Assumptions

The (1 + 1)-Evolution Strategy Sandpile model, described in detail in Section 6.1, was evaluated for its efficiency in localizing a contamination source using synthetic sensor data generated by the Gaussian plume model (Section  3).

The nature of these two models is similar. The Sandpile model is based on dropping sand grains onto a specific 2D point; the accumulation of grains causes avalanches, which consequently leads to a 3D structure. The advection mechanism introduced in our model causes the grains to fall not vertically but along a certain trajectory (see Figure 2 and Equation (2)). A model constructed in this way converges with the model of wind-borne substance transport in the air. The airborne substances consist of contamination particles, which were derived using the Gaussian plume model in this study. Therefore, there is significant similarity between the two models, enabling them to be mapped and compared effectively.

The concentrations reported by the sensor grid were fed into the (1 + 1)-ES algorithm to determine whether it could accurately identify an airborne contaminant source within the specified domain. The (1 + 1)-ES algorithm was applied to the Sandpile model, utilizing a two-dimensional cellular automaton (CA) of size 100×100. A larger CA size is expected to produce more accurate results, contingent upon a denser data grid. The number of sand grains ($G_{N}$) used in this model ranged from 0 to $10^{6}$ grains, while the grain size ($G_{s}$) varied from 0 to 1 meter. Wind direction and speed were represented by the vector $\left[w_{x},w_{y}\right]$. The algorithm ran for 200 generations.

In this context, an individual is represented by a pair of vectors (X, σ). The solution vector *X* is defined as $X\;=\;(X,Y,\;Z,w_{x},w_{y},\;G_{N},\;G_{s})$, while $\sigma \;=\;(\sigma_{X},\;\sigma_{Y},\;\sigma_{Z},\;\sigma_{w_{x}},\;\sigma_{w_{y}},\;\sigma_{G_{N}},\;\sigma_{G_{s}})$ represents the mutation step-size vector of the (1 + 1)-ES algorithm (explained in detail in Section 4). Here, X,Y, and *Z* denote the positions where grains of sand are dropped, $w_{x}$ and $w_{y}$ indicate the wind parameters, $G_{N}$ is the number of grains, and $G_{s}$ is the grain size. Each value of σ falls within the range [−5, 5]. Each experiment was conducted with 10 restarts.

## 7. Evaluation Results of the (1 + 1)-ES Sandpile Source Localization Model

Our research was structured in two sequential stages to address the problem’s complexity. The initial stage focused on an inverse problem: fitting the Sandpile model’s parameters to match the contaminant distributions reported by a network of sensors. This calibration process was essential to validate the model’s ability to accurately simulate real-world plumes. The second stage then leveraged this validated model to pinpoint the source characteristics, testing its efficacy by using the simulated plumes to reconstruct the original release location.

### 7.1. Adjusting the Parameters of the Sandpile Model to Align with Sensor Data

The first and most crucial stage involves experimentally adjusting the parameters of the Sandpile model to match the contaminant spread reported by the sensor network. A well-fitted Sandpile model will enable the identification of the release point in the second stage of the process.

**Figure 6:**
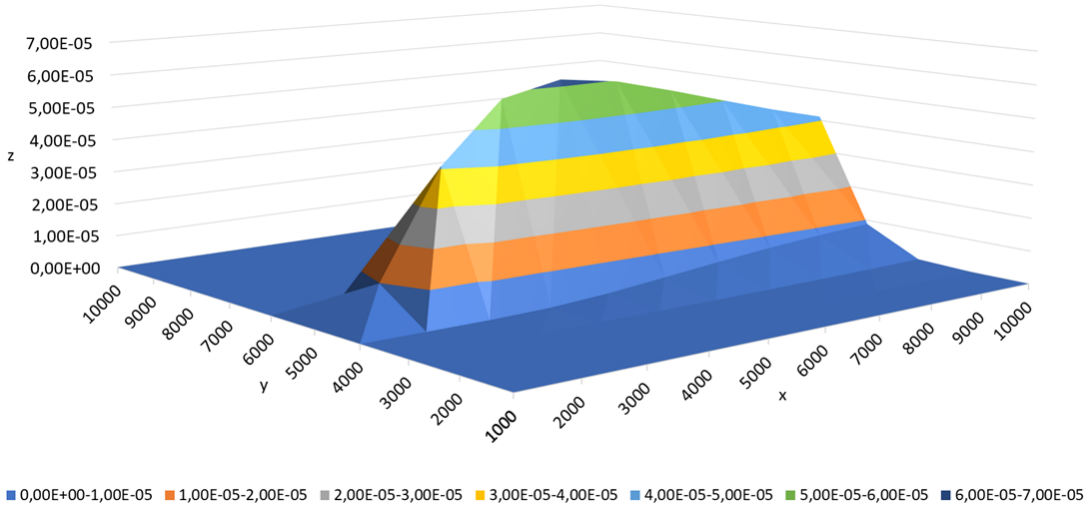
Three-dimensional distribution of the contaminant concentration for the first testing set with the following parameters: source position (0m,5000m,28.75m), emission rate of $5368.38\frac{g}{s}$, and wind directed along the *x*-axis.

Two groups of testing data were utilized. The first set was employed for preliminary tests (the first stage of experiments). This set comprised readings of contaminant concentration measured across a regular grid of sensors, with one sensor allocated to each square kilometer. The test domain was a square measuring 10 km by 10 km, containing 100 regularly spaced sensors arranged in a 1 km grid, positioned 2.5 m above the ground. The contaminant source was situated within the domain at the coordinates (0 m, 5000 m) and was 28.75 m above the ground. The emission rate was set at $Q=5368.38\;\frac{g}{s}$, with the wind blowing parallel to the x-axis at a speed of $5\;\frac{m}{s}$. A visualization of the simulation results from the Gaussian model contained in this dataset is shown in Figure 6.

In line with the assumptions and experimental plan, it was necessary to estimate the appropriate grain size and the correct number of grains to be dispensed. The results of the tests are presented in the tables below, considering all parameters of the Sandpile model.

The (1 + 1)-ES Sandpile model was executed to estimate the number of grains $G_{N}$ and the grain size $G_{s}$, while keeping the other parameters at the following levels: (X,Y) = (0, 50), Z = 28.75, and $\left[w_{x},w_{y}\right]=\left[1,\;0\right]$. The number of generations applied in the algorithm was equal to 200. The parameters tested and analyzed during the calibration of the Sandpile model to the Gaussian model included the following ranges: grain size $G_{s}$ within the range of [0, 100.0], gradient $\left[w_{x},w_{y}\right]=\left[1,\;0\right]$, number of grains $G_{N}$ within the range of $\left[1,\;10^{6}\right]$, and σ within the range of [−5.0, 5.0].

The results presented in Table 2 are sorted from the best fit to the worst.

The objective was to minimize the function $f(C_{j}^{M},C_{j}^{E})$, where a value of 0 indicates a perfect fit of the Sandpile model to the contaminant spread reported by the sensor network. As shown in Table 2, the best score was obtained with a grain size $G_{s}=0.21$ and a large number of grains $G_{N}=99,540$ (the first row in Table 2), yielding an evaluation function $f(C_{j}^{M},C_{j}^{E})=2727.47$. The last row of the table indicates that with a grain size that is too large—specifically, $G_{s}=16.72$—only a small number of grains were dispensed to fit the model, resulting in a poor fit (high evaluation function).

Despite the results obtained, the high value of the evaluation function indicates that the models did not achieve a good fit. Therefore, tests were conducted on the *Z*-coordinate, which had not been previously mapped to the simulation coordinates, such as the CA grid in the Sandpile model. Subsequent experiments were designed to determine the optimal height from which to pour the sand grains. The calibration was performed using the (1 + 1)-ES algorithm, which adjusted the calibrated values—either increasing or decreasing them—to achieve the best fit of the model to the measurement data provided by the Gaussian model. In these experiments, we analyzed *Z* within the range of [0,2000] and identified Z=1085 as the chosen value, with an evaluation function of $F(C_{j}^{M},C_{j}^{E})=1578.28$.

**Figure 7:**
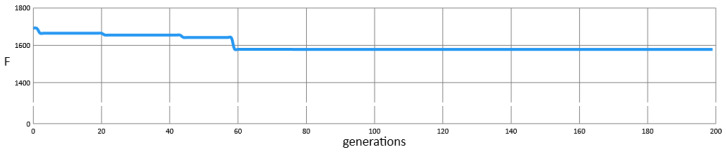
Algorithm run graph during the calibration of the Sandpile model to the Gaussian model, while finding our best approximation of both models $F(C_{j}^{M},C_{j}^{E})=1578.28$, using the following ranges: $G_{s}\in \left[0,\;100.0\right]$, gradient $\left[w_{x},w_{y}\right]=\left[1,\;0\right]$, number of grains $G_{N}\in \left[1,\;10^{6}\right]$ and σ∈[−5.0, 5.0].

Figure 7 illustrates changes in the fitness function throughout the iterations of calibrating the Sandpile model to match the Gaussian model. It can be observed that the algorithm achieved a result before the 60th generation, after four improvements.

A value as high as the obtained *Z*-coordinate may seem surprising at first. However, deposition height is intrinsically important and closely linked to grain size. In the Gaussian model, particles are in a state of continuous movement, influenced by wind force and direction. In contrast, the Sandpile model restricts a grain’s position to vertical changes (along the Z-coordinate) once it lands on the plane. When both the grain size is large and the deposition height is low, pile formation becomes impossible. In such cases, grains begin to accumulate in front of the intended pile beyond a certain point. The Sandpile model lacks mechanisms for pile displacement; thus, the only ways to change the pile’s location are through an avalanche (which causes a slope collapse) or by starting the pile in a different position. Therefore, maintaining an adequate deposition height is crucial in the Sandpile model to ensure the proper distribution of sand grains across the study area.

The initial phase of the experiments aimed to fit the Sandpile model to the sensor data in order to propose a dispersion model that closely aligns with the observed registrations. As a result, we selected the parameters that provided the best fit using the (1 + 1)-ES algorithm. The optimized Sandpile parameters were determined as follows: deposition height Z=1085, grain size $G_{s}=0.21$, and number of grains $G_{N}=99,540$.

### 7.2. Determining the Location of an Airborne Contaminant Source

**Figure 8:**
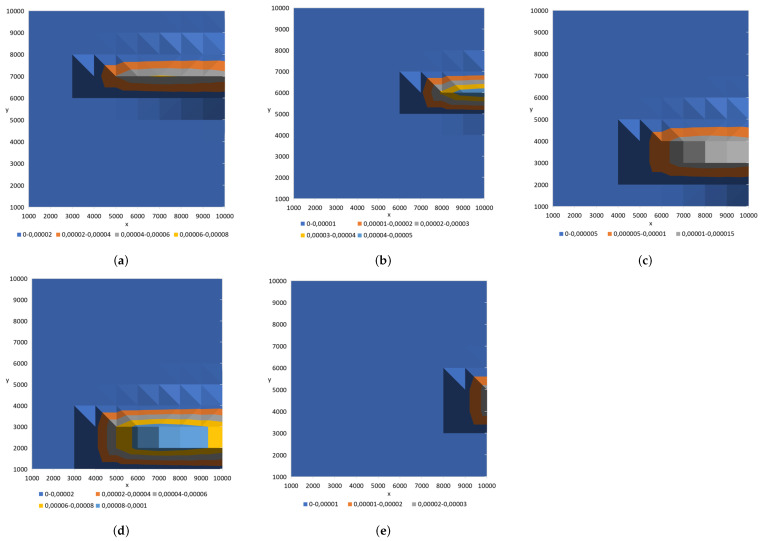
Contaminant distributions for five datasets used to evaluate the (1 + 1)-ES Sandpile algorithm in the context of contaminant source identification. Each subfigure (**a**–**e**) represents a different scenario modeled with a Gaussian distribution centered at the true source coordinates (in meters) (X,Y,Z): (**a**) Case 1: (2946,6824,29), (**b**) Case 2: (5744,6075,12), (**c**) Case 3: (3409,4119,25), (**d**) Case 4: (2455,2324,16), and (**e**) Case 5: (7878,4633,4).

The parameters of the Sandpile model selected in the previous stage of the algorithm were used to conduct tests on five different datasets. These datasets represented the concentration of the released substance in a sensor grid, with random source locations within the specified domain (see Figure 8).

In the datasets used in this section to evaluate the effectiveness of the proposed algorithm for contaminant source localization, the release height varied across scenarios (see Figure 8). As a result, the previously fixed sand-dumping height parameter, Z=1085 (see Section 7.1), was treated as a variable to be estimated alongside the horizontal source coordinates (X,Y,Z). The (1 + 1)-ES algorithm was employed in the following experiments to estimate the contaminant source position within the domain. The algorithm’s constant parameters were set as follows: grain size $G_{s}=0.21$, number of scattered grains $G_{N}=99.540$, and wind conditions $\left[w_{x},w_{y}\right]=\left[0,\;1\right]$. The objective was to accurately determine the source location (X,Y,Z). The analyzed parameters included the following ranges: grain size X,Y within the range of [0, 100], and *Z* within the range of [0, 2000]. All random processes utilized in this paper were executed using a system generator that applied a uniform distribution, with a seed based on the computer’s system clock. The results of these experiments are summarized in Table 3.

Table 3 presents a comparison between the true coordinates of the contaminant source (X,Y,Z) and the coordinates estimated by the (1 + 1)-ES Sandpile algorithm across five test cases. The algorithm performs well in estimating horizontal source locations, with most predicted *X* and *Y* values closely matching the true coordinates. Although some larger deviations appear, particularly in Cases 4 and 5, these fall within the expected accuracy range of approximately 1000 m, which corresponds to the sensor spacing in the input grid. This indicates that the observed horizontal errors are primarily due to grid resolution rather than limitations of the algorithm. In terms of vertical estimation, the algorithm tends to overestimate the release height. This behavior can be attributed to the intrinsic characteristics of the Sandpile model. In the Sandpile analogy, grains of sand are released from a specific height, with their size and number directly affecting the resulting distribution pattern. In contrast, when generating testing datasets of airborne contaminants using the Gaussian model, the physical weight of the particles is not explicitly considered in the same way. Consequently, the analogy between these two processes introduces a systematic bias in the estimated release height, making the *Z*-coordinate slightly less precise compared to the horizontal coordinates. The current results in this area were obtained through experimental methods. As mentioned in this paper, there seems to be a correlation between the grain deposition height in the Sandpile model and the release height of the hazardous substance during source localization. However, confirming this hypothesis requires considering additional factors, such as wind velocity and grain size within the Sandpile model, to formally establish this relationship through a mathematical equation. Establishing this dependency is expected to enhance the accuracy of localizing hazardous substance releases. Research in this area is ongoing, but it demands a substantial investment of time. In our specific case, errors along the *Z*-axis may be acceptable because we are examining the dispersion of a substance in an open area, where the primary goal is to determine the horizontal position of the source. Consequently, the release height is a secondary consideration. As shown in Table 3 and Table 4, there appears to be a correlation between the grain deposition height in the Sandpile model and the release height of the hazardous substance being localized. For the true coordinate *Z*, the source coordinates indicated by the (1 + 1)-ES Sandpile algorithm tend to grow consistently and quite proportionally across different experiments. Nonetheless, this trend is consistent across test cases, suggesting that it can be accounted for and potentially corrected in future refinements of the approach.

Overall, the (1 + 1)-ES Sandpile algorithm offers reliable and meaningful estimates of contaminant source locations, especially in the horizontal plane, making it a promising approach for environmental monitoring applications. The absolute errors for each coordinate are summarized in Table 4, which supports these observations. As can be seen from analyzing the data in Table 3 and Table 4, the Pearson correlation coefficient (*r*) for the fit of solutions obtained from the Sandpile–advection model and those generated by the Gaussian plume model is 0.9973 for the *X*-coordinate, 0.8898 for the *Y*-coordinate, and 0.6408 for the *Z*-coordinate. While the fit coefficients for the *X*- and *Y*-coordinates are relatively high—particularly for the *X*-coordinate—the coefficient for the *Z*-coordinate is considerably lower. This divergence can be attributed to the challenges involved in matching the grain size and pouring height, which results in significant differences, as discussed earlier in this chapter.

The variable number of grains between experiments is due to grains at the edge of the experimental area falling off. This loss of grains negatively impacts the results, especially when the symmetry of the model is disturbed. A greater number of fallen grains generally led to a lower correlation between the models. This trend is evident in Figure 8. Cases 1–3, which experienced minimal grain loss, demonstrate very high Pearson correlation coefficients (r=0.9996, 0.9994, and 0.9994, respectively). In Case 4, a moderate loss of grains with a disturbance of the symmetry in the model resulted in a significantly lower correlation coefficient (r=0.9650). Interestingly, Case 5 presented an anomaly: despite having the highest grain loss, its correlation coefficient (r=0.9913) was notably higher than that of Case 4, although it was still lower than those of Cases 1–3. This may be attributed to the preservation of the model’s symmetry, which allowed for a more accurate fit.

It is important to highlight the computational efficiency of the proposed algorithm. Each test case run on a standard laptop configuration was completed in under 20 min, which is significantly faster than many traditional inverse modeling approaches. This level of responsiveness is crucial in practical applications, where timely information is essential for effective decision-making. In emergency scenarios involving hazardous releases or environmental contamination, generating accurate source estimates within such short time frames can greatly improve the speed and effectiveness of response efforts. These results demonstrate the algorithm’s strong potential as a near-real-time solution for operational use in emergency management and environmental monitoring.

Our previous results using the Sandpile model without advection, optimized with the GEO algorithm [11], showed poor source localization performance. The average estimation error was 10% for the y-coordinate, but it increased dramatically to 200% for the x-coordinate due to the model’s inability to account for advection. Furthermore, applying a simple corrective shift using an empirically derived vector opposite to the wind improved the average estimation error to 10%, with many results often reaching around 5%. However, in the current approach, the average estimation error is 4%, with results frequently nearing 1%, and 5.5% for the y-coordinate.

The Layered Algorithm [5] identifies a probable source region rather than pinpointing an exact location in space. Its effectiveness is evaluated using three metrics: classification error, accuracy error, and relevance. Classification error refers to the ratio of the area identified by the algorithm as the probable source region to the total search area, expressed as a percentage. The classification error does not exceed 12% when using 10 sensors and decreases to less than 1% with 90 sensors. Accuracy error measures the Euclidean distance between the true source location and the geometric center of the sub-area designated by the algorithm. The average accuracy error decreases significantly as more sensors are added—from over 2000 m with 10 to 15 sensors down to 140 m with 100 sensors. Relevance is a binary metric (yes/no) that indicates whether the true source location falls within the designated sub-area. With just 20 sensors, the relevance is approximately 50% and increases almost linearly, reaching 100% when 100 sensors are utilized. The tests were conducted on a standard computer equipped with an Intel Core i7-8850H CPU at 2.60 GHz and 16 GB of RAM. The average computational time for data analysis using the Layered Algorithm in its sequential version was approximately 2.5 min for a single run with 10 sensors. In comparison, a single run with 100 sensors took nearly 10 min on average.

This performance is particularly noteworthy when compared to alternative approaches. For instance, the source reconstruction using Sequential Monte Carlo (SMC) and Bayesian methods described in [1] requires thousands of runs of the dispersion model to determine the probable characteristics of the source. Importantly, it achieves an accuracy that does not surpass that of our method. As a result, the high computational cost of these SMC-based techniques makes them impractical for real-time applications.

The optimization methods presented in this work can identify not only the source of airborne substances but also any substance measurable by detectors that indicate its accumulation (i.e., quantity or concentration). For example, [54] discusses a low-temperature plasma apparatus used for desorbing non-volatile analytes from porous surfaces. This technique can provide input data for the optimization model discussed in this paper, as well as in our previous research [5].

## 8. Conclusions

In a world facing the ongoing threat of airborne contaminants, this study introduces a novel approach to a critical issue: the rapid localization of an unknown source. By adapting the well-established Sandpile model, which is traditionally used to simulate self-organized criticality, we have demonstrated its potential as a computationally efficient tool for modeling atmospheric dispersion. This work successfully integrates the Sandpile model with the (1 + 1)-Evolution Strategy, creating a robust framework for solving the inverse problem of source term estimation. Using synthetic data generated by the established Gaussian dispersion model, our two-stage validation process has yielded highly promising results.

- Model Accuracy: We have demonstrated that our advection-enhanced Sandpile model accurately reproduces the contaminant concentration fields generated by the Gaussian model. This finding confirms that the simplified Sandpile approach can effectively capture the complex physics of atmospheric transport and dispersion. As shown in Figure 4, the heat maps of contaminant distribution from both models are nearly identical, validating the Sandpile model as a viable alternative to more complex simulations.
- Localization Performance: The (1 + 1)-ES Sandpile model proved to be effective at solving the inverse problem of source localization. By using only the sensor data as inputs, our proposed framework identified the source coordinates with acceptable accuracy. The algorithm’s ability to converge on the correct solution with minimal computational overhead highlights its superiority for emergency response applications, where time is critical. Our experiments demonstrated the model’s capability to accurately pinpoint the source, showcasing its practical utility for crisis management.
- Computational Efficiency: A key outcome of this research is the significant reduction in computational time compared to traditional multi-run dispersion models (e.g., [1]). Using the Sandpile model as a fast-forward simulator, we quickly iterated through potential solutions with the (1 + 1)-Evolution Strategy. This approach is well suited for real-time applications, where a rapid and accurate response is essential for public safety.

Future research should prioritize integrating more complex meteorological and environmental factors, including atmospheric turbulence, wind shear, and varied terrain, to enhance the model’s accuracy.

## Figures and Tables

**Table 1:** The Pasquill–Gifford stability classes defined based on meteorological conditions [49,50,51,52].

Surface Wind	Daytime Incoming Solar Radiation			Nighttime Cloudiness	
Speed (m/s)	Strong	Moderate	Slight	>4/8	≤3/8
<2	A	A–B	B	E	F
3	A–B	B	C	E	F
4	B	B–C	C	D	E
5	C	C–D	D	D	D
>6	C	D	D	D	D

**Table 2:** Summary of the optimal configurations for the grain number $G_{N}$ and grain size $G_{s}$, as determined by the (1 + 1)-ES Sandpile algorithm. The results were obtained under the following constants: (X,Y)=(0,50), Z=28.75m, $\left[w_{x},w_{y}\right]=\left[1,0\right]$, and a total of 200 generations.

Lp.	X	Y	Z	$\left[w_{x},w_{y}\right]$	$G_{s}$	$G_{N}$	$f(C_{j}^{M},C_{j}^{E})$
1	0	50	28.76	[1, 0]	0.21	99,540	2727.47
2	0	50	28.76	[1, 0]	0.19	96,956	2727.52
3	0	50	28.76	[1, 0]	0.05	77,044	2737.96
4	0	50	28.76	[1, 0]	0.01	72,947	2763.87
5	0	50	28.76	[1, 0]	0.05	67,179	2825.11
6	0	50	28.76	[1, 0]	0.06	50,064	2903.14
7	0	50	28.76	[1, 0]	0.24	42,460	2978.77
8	0	50	28.76	[1, 0]	0.28	22,985	3093.93
9	0	50	28.76	[1, 0]	0.01	11,976	3106.10
10	0	50	28.76	[1, 0]	16.72	517	3325.24

**Table 3:** Comparison of the true release source coordinates within the domain for the testing datasets, along with the corresponding source coordinates estimated by the Sandpile model and the (1 + 1)-ES algorithm.

Lp	True Source Coordinates			Source Coordinates Indicated by the (1 + 1)-ES Sandpile Algorithm		
	X	Y	Z	$X_{\mathrm{est}}$	$Y_{\mathrm{est}}$	$Z_{\mathrm{est}}$
1	2946	6824	29	3000	6900	365
2	5744	6075	12	5800	5900	201
3	3407	4119	25	3400	3900	295
4	2455	2324	16	2900	3600	507
5	7878	4633	4	7800	3700	21

**Table 4:** Absolute errors between true source coordinates and those estimated by the (1+1)-ES Sandpile algorithm.

Lp	True Coordinates			Absolute Errors		
	X	Y	Z	$|X_{\mathrm{est}}-X|$	$|Y_{\mathrm{est}}-Y|$	$|Z_{\mathrm{est}}-Z|$
1	2946	6824	29	54	76	336
2	5744	6075	12	56	175	189
3	3407	4119	25	7	219	270
4	2455	2324	16	445	1276	491
5	7878	4633	4	78	933	17

## Data Availability

The raw data supporting the conclusions of this article can be made available by the authors on request.
